# *Cap’n’collar* differentiates the mandible from the maxilla in the beetle *Tribolium castaneum*

**DOI:** 10.1186/2041-9139-3-25

**Published:** 2012-11-01

**Authors:** Joshua F Coulcher, Maximilian J Telford

**Affiliations:** 1 Department of Genetics, Environment and Evolution, University College London, Darwin Building, Gower Street, London, WC1E 6BT, UK

**Keywords:** Beetle, *cap’n’collar*, *Deformed*, Endite, Labrum, Mandible, Maxilla, RNAi, *Tribolium*

## Abstract

**Background:**

The biting mandible of the arthropods is thought to have evolved in the ancestor of the insects, crustaceans and myriapods: the Mandibulata. A unique origin suggests a common set of developmental genes will be required to pattern the mandible in different arthropods. To date we have functional studies on patterning of the mandibular segment of *Drosophila melanogaster* showing in particular the effects of the gene *cap’n’collar* (*cnc*), however, the dipteran head is far from representative of insects or of more distantly related mandibulates; *Drosophila* does not even possess a mandibular appendage. To study the development of a more representative insect mandible, we chose the red flour beetle *Tribolium castaneum* and investigated the function of the *Tribolium* orthologs of *cap’n’collar* (*Tc-cnc*) and the Hox gene *Deformed* (*Tc-Dfd*). In order to determine the function of *Tc-cnc and Tc-Dfd,* transcripts were knocked down by maternal RNA interference (RNAi). The effects of gene knockdown were examined in the developing embryos and larvae. The effect of *Tc-cnc* and *Tc-Dfd* knockdown on the expression of other genes was determined by using *in situ* hybridization on *Tribolium* embryos.

**Results:**

Our analyses show that *Tc-cnc* is required for specification of the identity of the mandibular segment of *Tribolium* and differentiates the mandible from maxillary identity. Loss of *Tc-cnc* function results in a transformation of the mandible to maxillary identity as well as deletion of the labrum. *Tc-Dfd* and the *Tribolium* homolog of *proboscipedia* (*Tc-mxp* = *maxillopedia*)*,* Hox genes that are required to pattern the maxillary appendage, are expressed in a maxilla-like manner in the transformed mandible. *Tribolium* homologs of *paired* (*Tc-prd)* and *Distal-less (Tc-Dll)* that are expressed in the endites and telopodites of embryonic appendages are also expressed in a maxilla-like manner in the transformed mandible.

We also show that *Tc-Dfd* is required to activate the collar of *Tc-cnc* expression in the mandibular segment but not the cap expression in the labrum. *Tc-Dfd* is also required for the activation of *Tc-prd* in the endites of the mandible and maxillary appendages.

**Conclusions:**

*Tc-cnc* is necessary for patterning the mandibular segment of *Tribolium*. Together, *Tc-cnc* and *Tc-Dfd* cooperate to specify mandibular identity, as in *Drosophila.* Expression patterns of the homologs of *cnc* and *Dfd* are conserved in mandibulate arthropods suggesting that the mandible specifying function of *cnc* is likely to be conserved across the mandibulate arthropods.

## Background

The arthropod mandible is an appendage adapted for biting and chewing and is present in three arthropod groups, the insects and crustaceans (collectively the Pancrustacea) and the myriapods (millipedes and centipedes). The mandibulate arthropods, commonly grouped together in the monophyletic Mandibulata, constitute the majority of animals both in terms of numbers of species and biomass on this planet. The mandible is therefore an evolutionary novelty of particular interest.

There are many different types of mandible, but the characteristic that most mandibles share, and which differentiates it from other arthropod appendages, is the presence of a functional biting edge made up of the incisor and molar processes. This gnathal edge is widely considered to be a homologous structure within the Mandibulata [1-3].

Other arthropod groups, the chelicerates and trilobites, do not have mandibles and instead have a walking leg on the homologous segment to the mandibular segment [4,5]. An unsegmented appendage, or lobopod, is present in closely related outgroups to the arthropods, such as the onychophorans and tardigrades [6].

An alternative phylogenetic hypothesis to the monophyletic Mandibulata is the Myriochelata hypothesis, which groups the myriapods with the chelicerates. Accepting this hypothesis would suggest that the mandible evolved independently in the Myriapoda and Pancrustacea or that it has reverted to a walking leg in the Chelicerata [7]. While still controversial, recent molecular phylogenies including evidence from unique microRNAs favour Mandibulata over Myriochelata. This phylogeny is also strongly supported on morphological grounds [8-11].

### Mandible evolution

The mandible is serially homologous with other arthropod post-antennal appendages all of which are thought to have evolved from a segmented biramous limb. The archetypal biramous limb consists of a protopodite (the base of the limb) to which are attached two branches: the telopodite (or palp) and an exopodite [12-14]. Structures called endites, often involved in food processing, are also present on the protopodite. The gnathal edge of the mandible is thought to have evolved from the proximal most endite on the protopodite of this ancestral biramous limb [2,11]. The mandible is thought to be a gnathobasic structure and this interpretation is supported by expression data: the distal limb expression domain of *Distal-less (Dll)* is missing from the embryonic mandibular limb bud in diverse mandibulate arthropods [15-17].

All arthropod mandibles appear to be gnathobasic and are restricted to a monophyletic group implying that the mandible has a unique origin and is a homologous structure between mandibulate arthropods. We might therefore expect significant similarities in the embryonic patterning of the mandible between diverse mandibulate taxa. Finding the identity of the genes that pattern the mandible and showing how they function in diverse arthropod taxa could support the view that the mandible is homologous across the Mandibulata and, through comparisons with non-mandibulate sister groups, could give an insight into how the mandible evolved from a primitive arthropod limb.

We have undertaken a functional study of some of the genes that pattern the mandible in a model organism with a typical insect mandible to compare its development with the development of mandibles in other taxa. We chose to study the red flour beetle *Tribolium castaneum* that, unlike *Drosophila melanogaster*, has a canonical mandible in which the gnathal edge is made up of the incisor and molar processes.

### Mandibular segment patterning in *Drosophila*

The majority of research into the function of genes patterning arthropod gnathal appendages has focused on insects with very derived mouthparts, in particular the involuted larval head and non-biting adult proboscis of the dipteran *D. melanogaster*[18-25] and the stylet of the hemipteran *Oncopeltus fasciatus*[26,27].

Although developing *Drosophila* embryos possess gnathal lobes (structures from which the gnathal appendages are formed in other less derived insects [28]), following head involution, *Drosophila* larvae do not have any gnathal appendages [28-31] and both larval and adult *Drosophila* lack an appendage on the mandibular segment.

In *Drosophila,* the gene *Deformed* (*Dfd*) is required for the specification of both mandibular and maxillary identities [23-25,32,33]. *Dfd* does not differentiate the mandibular segment from the maxillary segment; for this function another gene, *cap’n’collar* (*cnc*)*,* is required [22-24]. *cnc* is a basic leucine zipper family gene (bZIP) that is expressed in an anterior ‘cap’ domain in the labrum and a posterior ‘collar’ domain in the mandibular segment and is necessary for the development of both labral and mandibular derived structures. It is likely that *cnc* achieves its mandible patterning function in part indirectly by repressing the maxilla patterning function of *Dfd: Dfd* expression is repressed by *cnc* in the anterior of the mandibular gnathal lobe and the activity of the Dfd protein is also repressed by *cnc* in the mandibular segment. *cnc* null mutants lose both labral and mandibular segment derived structures and have a duplication of maxillary structures [22-24,34].

### Previous work in *Tribolium*

In *Tribolium,* Brown *et al.* have demonstrated that the homolog of *Dfd, Tc-Dfd,* is necessary for patterning the mandibular and maxillary segments and that *Tc-Dfd* expression is progressively downregulated in the mandibular limb buds as in *Drosophila*[35,36]. In *Tc-Dfd* mutants there is a homeotic transformation of the mandible to an antenna and a loss of the maxillary endites. *Dfd,* although required for mandible development, does not differentiate the mandibular segment from the maxillary segment in *Drosophila* or *Tribolium*. The role of *Tc-cnc* in *Tribolium* is not known, however, it is expressed in a very similar pattern to that seen in *Drosophila*[37] and this is also true of *cnc* in other mandibulate arthropods [38-40] suggesting it may have a conserved function. Embryonic expression in non-mandibulate arthropods is not known.

### Experimental outline

With the ultimate aim of understanding the origin of the mandible, we were interested in the role that *Tc-cnc* might play in patterning the mandibular segment of *Tribolium castaneum,* a mandible-bearing insect. In order to test its function in *Tribolium*, *Tc-cnc* was knocked down using parental RNA interference (RNAi) by injecting double-stranded RNA (dsRNA) into female *Tribolium* pupae [41]. The knockdown phenotype was detected both in embryos and in the first instar larvae of offspring of injected parents. The effect of *Tc-cnc* knockdown on downstream genes was studied by *in situ* hybridization in *Tribolium* embryos.

## Methods

### *Tribolium castaneum* culture

Wild-type *T. castaneum* (San Bernardino strain) were kindly provided by Dr Gregor Bucher (Department of Developmental Biology, Georg-August-University Göttingen, Göttingen, Germany) and raised at 32°C in organic wholemeal flour supplemented with 5% brewer’s yeast.

### Cloning of *Tribolium* orthologs

*Tc-cnc, Tc-Dfd, Maxillopedia* the *Tribolium* ortholog of *Proboscipedia* (*Pb*) (*Tc-mxp*)*,* the *Tribolium* ortholog of *paired* (*Tc-prd*) and *Tc-Dll* were amplified from mixed stage cDNA by polymerase chain reaction (PCR) amplification using the following primers: *Tc-cnc,* a 2,612 bp clone for hapten-labelled RNA probe synthesis (forward: 5^′^-GCAACAGTGGGCCCTATTTA-3^′^ and reverse: 5^′^-GTGGTGGCTCCTTGTGTTCT-3^′^). *Tc-cnc,* a 633 bp clone for dsRNA synthesis (forward: 5^′^-GATTACAGCTATACGAGTCGG-3^′^ and reverse: 5^′^-GTCAGCCAGACTCAAAATCTG-3^′^). *Tc-Dfd* (forward: 5^′^-CCAAGTGAGGAGTACAACCAG-3^′^ and reverse: 5^′^-TACAAGGCCGTGAGTCCGTAA-3^′^), *Tc-mxp* (forward: 5^′^-ATAGCTGCTTCGCTAGACCTTA-3^′^ and reverse: 5^′^-TCGCAGGTGGGGTCATTAT-3^′^), *Tc-Dll* (forward: 5′-CAGCAGGTGCTCAATGTGTT-3^′^ and reverse: 5^′^-ATTAAACAGCTGGCCACACC-3^′^), *Tc-prd* (forward: 5^′^-ATGCACAGACATTGCTTTGG-3^′^ and reverse: 5^′^-GGATCGTCACAGTGTTGGTG-3^′^). Accession numbers are as follows: *Tc-cnc* (GenBank: NM_001170642.1), *Tc-Dfd* (GenBank: NM_001039421), *Tc-mxp* (GenBank: NM_001114335), *Tc-Dll* (GenBank: NM_001039439), *Tc-prd* (GenBank: NM_001077622).

### Parental RNAi

Parental RNAi was performed as previously described [41]: 0.25 to 0.4 μl of *Tc-cnc* dsRNA (dissolved in distilled water at a concentration of 0.36 to 3 μg/μl) was injected into female pupae. Then, 633 bp of *Tc-cnc* dsRNA (positions 1,389 to 2,021, including part of the bZIP domain which starts at position 1,932) was injected. Embryos were either fixed 24 to 48 h after egg laying or left to develop into first instar larvae for cuticle preparation. In total, 1,736 female beetle pupae were injected for collecting embryos for *in situ* hybridization.

In order to characterize the *Tc-cnc* phenotype, 218 female pupae were injected with 1 to 2 μg/μl dsRNA and the cuticles of first instar larvae were analyzed. Of these 218 injected pupae, 195 successfully eclosed. At 20 days after injection a further 117 beetles (60%) had died. Parental injection of *Tc-cnc* dsRNA resulted in the mortality of a much greater number of injected females compared to the numbers killed in other RNAi experiments, in which typically 10% of injected female beetles die by day 20 (data not shown). The higher mortality rate may be a consequence of the effects of *Tc-cnc* knockdown. Only one phenotype was detected in first instar larvae: transformation of the mandible to maxillary identity and loss of the labrum.

In order to obtain partial phenotypes (incomplete transformations of the mandible to maxillary identity) we tried injecting lower concentrations of *Tc-cnc* dsRNA (360 to 750 ng/μl). However, only wild-type larvae or those with fully transformed mandibles were obtained, and no partial phenotypes were observed. Similar rates of mortality were observed even at lower concentrations.

To obtain *Tc-Dfd*^*RNAi*^ embryos, 1,142 bp (positions 491 to 1,632) *Tc-Dfd* dsRNA was injected into female pupae and embryos were fixed for *in situ* hybridization. The *Tc-Dfd*^*RNAi*^ phenotype was confirmed by comparing cuticle preparations of first instar larvae to previously described phenotypes [36,42].

### Cuticle preparation

Cuticles from first instar larvae were prepared in Hoyer’s medium and lactic acid as previously described [43]. The cuticle preparations were observed using differential interference contrast (DIC) and confocal fluorescent microscopy (larval cuticle autofluoresces at visible wavelengths). Cuticle preparations were observed using confocal microscopy with an excitation frequency of 488 nm using an upright Leica TCS SPE confocal microscope (Leica microsystems, Wetzlar, Germany). Images were obtained and edited using Leica application suite advanced fluorescence software, LAS-AF (Leica microsystems, Wetzlar, Germany).

### Whole mount *in situ* hybridization

Embryos were fixed in 9% formaldehyde. Both single stainings (nitro blue tetrazolium/5-bromo-4-chloro-3-indolyl phosphate (NBT/BCIP)) and double stainings (NBT/BCIP and FastRed) were performed as previously described [44]. Some modifications, for example in the frequency and duration of washes, were incorporated from alternative *in situ* hybridization protocols [45].

Stained embryos were dissected from their yolk and mounted in glycerol. Embryos (and cuticle preparations) were observed using differential interference contrast (DIC) microscopy with an Imager M1 microscope (Carl Zeiss Ltd., Cambridge, UK). Images were taken with Axiocam HRC (Carl Zeiss Ltd., Cambridge, UK) and processed using Axiovision product suite software release 4.8.2 (Carl Zeiss Ltd., Cambridge, UK). Images were edited with GIMP (release 2.6.10.) [46].

### Scanning electron microscopy

Embryos were fixed as described for the whole mount *in situ* hybridization protocol. Fixed embryos were rinsed in ethanol and immersed in hexamethyldisilazane (HMDS), air dried and sputter coated with gold. Images were taken in a JEOL JSM-5410LV scanning microscope **(**JEOL Ltd., Tokyo, Japan) at a magnification of 100 to 350 fold and processed with DigitalMicrograph (Gatan Inc., Pleasanton, California, USA).

## Results

### *Tc-cnc* expression

**Figure 1:**
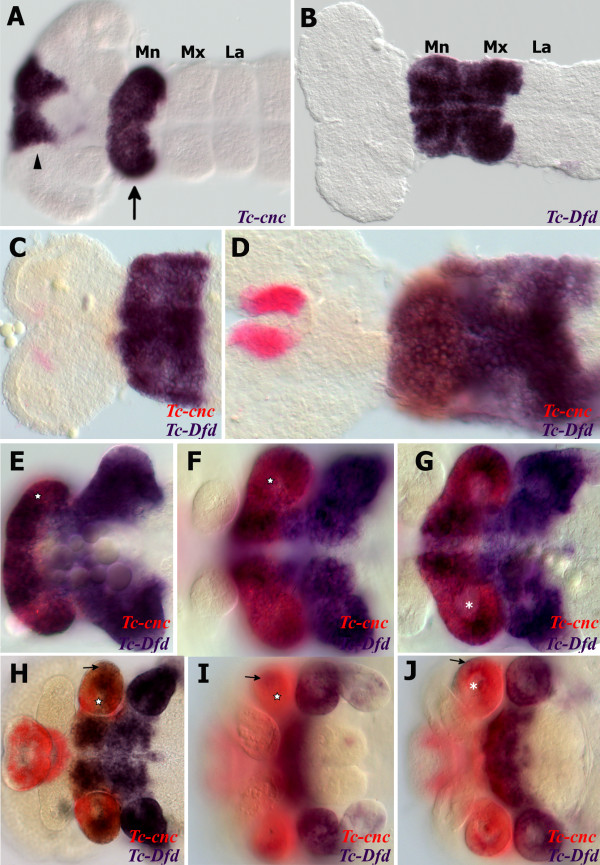
**Expression of *Tc-Dfd *and *Tc-cnc *in the mandibular and maxillary segments.** All views are ventral with anterior to the left unless otherwise indicated. Gene expression was determined by *in situ* hybridization. (**A**) Expression of *Tc-cnc* in a germ band extending stage embryo. There is an anterior cap domain of *Tc-cnc* in the labrum (arrowhead). The posterior collar domain is present in the mandibular segment (arrow). (**B**) *Tc-Dfd* expression in a germ band extending embryo as limb buds are just about to form. Expression is present throughout the mandibular and maxillary segments. (**C-J**) Expression of *Tc-Dfd* (blue) and *Tc-cnc* (red) in wild-type embryos. Coexpression of *Tc-Dfd* and *Tc-cnc* is brown. (**C**) Early germ band extending embryo. (**D**) Germ band extending stage embryo prior to limb bud formation. (**E**) Germ band extending embryo. (**F**) Late germ band extending embryo. (**G**) Same embryo as (**F**), but a lower plane of focus that shows the reduction of *Tc-cnc* expression in the mesoderm (asterisk). (**H**) Germ band retracting embryo. (**I**) Embryo undergoing dorsal closure with the gnathal appendages moving towards the ventral midline. (**J**) Same embryo as (**I**), but a lower plane of focus that shows the reduction of *Tc-cnc* expression in the mesoderm (asterisk). (**C**,**D**) Prior to limb bud formation, *Tc-Dfd* expression is continuous throughout the mandibular segment. (**E**,**F**) As soon as the endites start to form, *Tc-Dfd* expression retracts from the developing mandibular endites (star) whilst *Tc-cnc* expression is maintained throughout the mandibular appendage. (**G**-**J**) By late embryogenesis, faint *Tc-Dfd* expression is only present in the lateral part of the mandibular limb bud (arrow), and missing from the ventral-medial region (star). *Tc-Dfd* expression is still strongly maintained in the maxillary limb bud. Mandibular (Mn), maxillary (Mx) and labial (La) segments.

*Tc-cnc* is expressed in two distinct domains, an anterior cap that includes the developing labrum and around the stomodeum and a posterior collar domain in the mandibular segment (see Figure 1A) [37]. *Tc-cnc* expression remains constant in these two domains from their first appearance during germ band elongation and through late embryogenesis (see Figure 1D,F,H) and is expressed in regions of the mandibular limb bud where *Tc-Dfd* expression becomes repressed (see star in Figure 1E-I). In the mandibular limb bud, *Tc-cnc* is expressed predominantly in the ectoderm, with weaker expression (or no discernable expression) in the mesoderm of the limb bud (see asterisk in Figure 1G,J).

### *Tc-Dfd* expression retracts from the developing mandible

*Tc-Dfd* is expressed throughout the mandibular and maxillary segments in the early developing *Tribolium* embryo (see Figure 1B). As the mandibular limb buds start to form, *Tc-Dfd* expression progressively retracts from the ventral-proximal region of the mandibular limb bud (see Figure 1E-J). *Tc-Dfd* continues to retract from this ventral-proximal region (star in Figure 1E).

**Figure 2:**
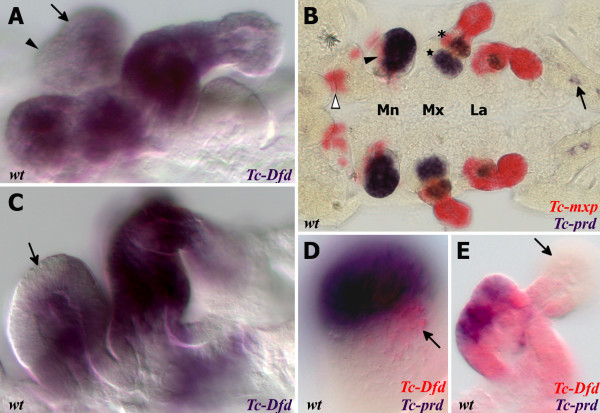
**Expression of *Tc-Dfd, Tc-mxp *and *Tc-prd *in dissected embryonic mandibles and maxillae.** All views are ventral with anterior to the left unless otherwise indicated. Gene expression was determined by *in situ* hybridization. (**A,C**) Lateral view of the mandibular and maxillary appendages showing *Tc-Dfd* expression (blue) in a germ band fully retracted stage embryo. (A) *Tc-Dfd* expression is repressed from the developing mandibular endite, which consists of an inner lobe (arrowhead) and an outer lobe (arrow). (**B**) Embryo stained with *Tc-mxp* (red) and *Tc-prd* (blue). *Tc-mxp* is expressed in the maxillary and labial palps and the distal protopodite of both appendages. In the maxilla, protopodite expression relates to the position of the developing galea endite lobe (asterisk), which is marked by the distal domain of *Tc-prd* expression. *Tc-mxp* is expressed in the mesoderm of the mandibular limb bud (arrowhead). The intercalary domain of *Tc-mxp* expression is also visible (white arrowhead). Mesodermal expression of *Tc-prd* is present in the telopodites of post-antennal appendages but clearly visible in the developing leg appendages (arrow). (**C**) *Tc-Dfd* expression is missing from the outer lobe of the mandible (arrow). (**D,E**) *Tc-Dfd* expression (red) and *Tc-prd* expression (blue) in a dissected mandible and maxilla of a post germ band retracted stage embryo undergoing dorsal closure. Distal is top. (**D**) Lateral view of a dissected mandible. *Tc-Dfd* expression remains on the lateral side of the mandible (arrow). (**E**) Dissected maxilla, lateral is to the right. *Tc-Dfd* expression is throughout the protopodite and at the base of the palp. The distal part of the palp is lacking or has weak *Tc-Dfd* expression (arrow).

In the developing maxillae, *Tc-Dfd* expression is continually expressed in the protopodite (see Figure 1E-J). Mandibular *Tc-Dfd* is increasingly repressed until only weak expression remains on the lateral side of the mandible (see Figure 1H,I and Figure 2A,C,D).

The mandibular limb bud has two lobes, the inner and the outer (see Figure 2A). The distal-most part of the mandibular limb bud becomes the outer lobe of the mandible and develops into the future incisor process. *Tc-Dfd* is not present in this most distal region, which is more clearly noticeable in lateral orientations of dissected *Tribolium* embryos (see Figure 2C).

We found that *Tc-prd*, in addition to its function as a secondary pair-rule gene [47], is expressed in the predicted location of the developing endites of the embryonic mandibular, maxillary and labial limb buds (see Figure 2B) [48]. We therefore used *Tc-prd* expression as a marker for endite development. *Tc-prd* expression reveals that the ventral-medial region of the mandibular limb bud, where *Tc-Dfd* expression is lost, encompasses the mandibular endite and the immediate surrounding tissue. *Tc-Dfd* expression is retained in the lateral part of the mandibular limb bud, but fades throughout embryogenesis (Figure 2D). *Tc-Dfd* expression is absent (or considerably weaker) in the distal part of the maxillary palps throughout embryogenesis (see arrow in Figure 2E).

### *Tc-cnc* RNAi phenotype

**Figure 3:**
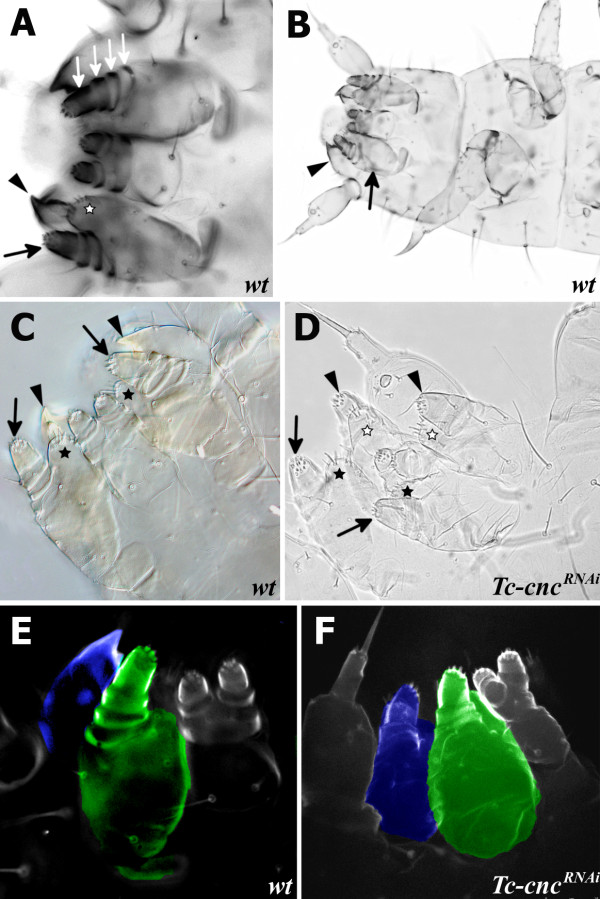
***Tc-cnc***^***RNAi***^**results in transformation of the mandible into maxillary identity.** Mandible (arrowhead), maxillary palp (arrow) and maxillary ventral branch (star) are indicated on cuticle preparations of wild-type and *Tc*-*cnc*^*RNA*^*Tribolium* first instar larvae. (**A**) Cuticle preparations of gnathal appendages visualized by fluorescence microscopy. The maxillary appendages have a palp with four segments (white arrows) attached to a protopodite with the maxillary endites (lacinia and galea) that, in first instar larvae, are fused to form the ventral branch (star). (**B**) Cuticle preparation of a first instar *Tribolium* larva. (**C**) Cuticle preparation of the larval gnathal appendages of a wild-type *Tribolium* larva visualized by DIC microscopy. (**D**) Cuticle preparation of the gnathal appendages of a *Tc-cnc*^*RNAi*^ larva. Knockdown of *Tc-cnc* results in transformation of the mandibular appendages into maxillary appendages (arrowheads). The ventral branch is visible on the transformed appendages (white stars). The maxillary appendage is indicated with arrows (palp) and black stars (ventral branch). (**E**) Cuticle preparation of wild-type *Tribolium* larva visualized by confocal microscopy. The mandibular appendage is highlighted in blue; the maxillary appendage is highlighted in green. (**F**) Cuticle preparation of a *Tc-cnc*^*RNAi*^ larva visualized by confocal microscopy. The transformed mandibular appendage is highlighted in blue and clearly resembles the maxillary appendage (highlighted in green).

In order to test the role *Tc-cnc* might play in patterning the mandibular segment, the gene was knocked down in developing embryos by injecting *Tc-cnc* dsRNA into female pupae. The knockdown phenotype was determined in the offspring of injected parents using cuticle preparations of their first instar larvae (see Figure 3).

**Figure 4:**
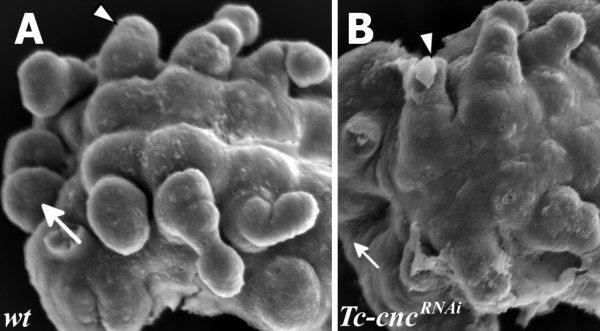
***Tc-cnc***^***RNAi***^**results in deletion of the Labrum.** Scanning electron micrographs (SEMs) of wild-type and *Tc*-*cnc*^*RNA*^ embryos shows the deletion of the Labrum in *Tc-cnc*^*RNAi*^ embryos. All views are ventral with anterior to the left. (**A**) SEM of a wild-type embryo at fully extended germ band stage. The labral buds are clearly visible at the anterior of the embryo (arrow). The mandible is indicated (arrowhead). (**B**) SEM of *Tc-cnc*^*RNAi*^ embryo at germ band extending stage. The labral buds are missing (arrow). The mandible is transformed into maxillary identity (arrowhead).

Injection of *Tc-cnc* dsRNA produces phenotypes that relate to both the cap domain and the collar domain of *Tc-cnc* expression. The effect in the collar domain is the homeotic transformation of the mandibular appendage into a maxillary identity showing that the posterior collar domain of *Tc-cnc* expression differentiates the mandible from the maxillary appendage. This is shown in Figure 3D,F, where *Tc-cnc*^*RNAi*^ larvae can be seen to possess an additional pair of maxillae. The mandibular appendages are transformed into a maxillary identity, in possession of a maxillary palp, and maxillary endites (which in wild-type first instar *Tribolium* larvae are fused to form the ventral branch; see Figure 3A,C). Knockdown of the cap domain results in a dramatic deletion of the labrum showing *Tc-cnc* is necessary to pattern this structure (see Figure 4B). There are also abdominal defects visible in some embryos, although it is possible that this aspect of the phenotype was an artifact of the cuticle preparation procedure.

### *Tc-cnc* represses *Tc-Dll* and modifies *Tc-prd* expression in the Mandibular segment

**Figure 5:**
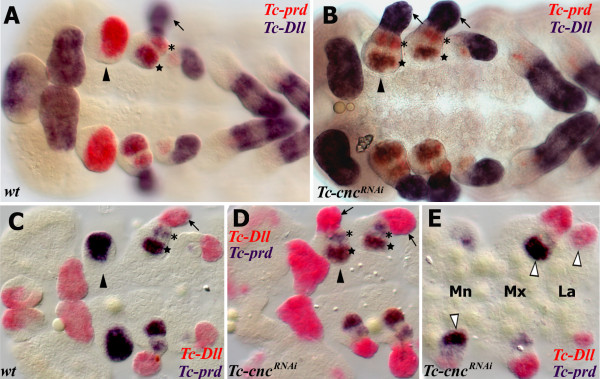
**Homeotic transformation of the mandibular appendage to maxillary identity in *Tc-cnc *knockdown embryos as revealed by the expression of markers for telopodites (*Tc-Dll*) and endites (*Tc-prd*)*.*** Gene expression was determined by *in situ* hybridization. All views are ventral with anterior to the left. (**A-E**) The mandibular segment appendage (arrowhead), lacinia (star), galea (asterisk) and telopodite (arrow) are indicated. (**A**,**B**) Expression of *Tc-Dll* (blue) and *Tc-prd* (red). (**A**) wild-type embryo. *Tc-Dll* is expressed in the maxillary lacinia endite lobe (star) and telopodite (arrow). *Tc-prd* is expressed in the endites of the mandible, maxilla and labial appendages. (**B**) *Tc-cnc*^*RNAi*^ embryo. *Tc-Dll* and *Tc-prd* are expressed in transformed mandible appendages in the same manner as in the maxillae. The labral domain of *Tc-Dll* is also missing at the anterior of the embryo. (**C**-**E**) Expression of *Tc-Dll* (red) and *Tc-prd* (blue) in germ band extending embryos earlier than those shown in **A**,**B**. (**C**) wild-type embryo. (**D**,**E**) *Tc-cnc*^*RNAi*^ embryos. (**E**) *Tc-cnc*^*RNAi*^ embryo*.* The telopodites and endites of some appendages are larger (white arrowheads) than the corresponding appendage on the other side of the same segment. There is an asymmetry between the different transformed mandibular appendages. The transformed mandibles resemble maxillae at an earlier stage of development and so have delayed development relative to the maxillary appendages. Mandibular (Mn), maxillary (Mx) and labial (La) segments shown.

To investigate the transformed mandibular appendage in *Tc-cnc* knockdown embryos, the expression patterns of the homeobox genes *Tc-prd* and *Tc-Dll* were studied as genetic markers of the developing endites and telopodites respectively (see Figure 5).

In wild-type embryos, *Tc-prd* is expressed in the developing endites of all three pairs of gnathal appendages (mandibles, maxillae and labia; see Figure 2B and Figure 5A,C). There are two distinct domains of *Tc-prd* expression in the maxilla, which we assume correspond to the developing lacinia and galea. There is a single domain of *Tc-prd* in the labial appendage and a larger single domain of expression in the mandibular appendage.

*Tc-Dll* is expressed in the distal part of all appendages of wild-type *Tribolium* embryos except the mandible. In the developing maxilla, there are two domains of *Tc-Dll* expression, a distal domain in the developing palp and a proximal domain in the lacinia endite. *Tc-cnc* RNAi results in homeotic transformation of the mandibular appendage into maxillary identity. The solitary domain of *Tc-prd* expression in the mandible is transformed into two domains of *Tc-prd* expression that relate to the maxillary endites (see Figure 5B,D-F). *Tc-Dll* is de-repressed resulting in expression in the palp and in a proximal endite that appears on the transformed mandible.

The transformed mandibular appendage develops more slowly than the adjacent true maxillary appendages at several stages of embryogenesis resembling the maxillary appendage of an earlier stage (see Figure 5E). By late embryogenesis, there is no evident morphological difference between the maxillae and the ectopic maxillary appendages on the mandibular segment.

Asymmetry of different appendages in *Tc-cnc* RNAi embryos is often evident in germ band extending stage embryos and occurs left or right at random (see Figure 5E). This does not appear to be an artifact of the RNAi procedure or the *in situ* hybridization process as appendages other than the mandible can be affected and parental RNAi experiments of other genes in *Tribolium* have not yielded a similar result (data not shown). Instead this may be related to a loss of the role that *cnc* has been shown to have in *Drosophila* in protecting the embryo from oxidative stress [49].

### *Tc-Dfd* and *Tc-mxp* are expressed in a maxilla-like manner in the transformed mandibular limb bud of *Tc-cnc*^*RNAi*^ embryos

The Hox genes *Tc-Dfd* and *maxillopedia (Tc-mxp),* the *Tribolium* ortholog of *pb,* pattern the maxillary appendage in an additive fashion. *Tc-Dfd* is expressed in the proximal part of the maxilla (the protopodite), and *Tc-mxp* is expressed in the palp and is excluded from the proximal part of the protopodite, although it is expressed in the distal protopodite and galea endite (see Figure 2B). *Tc-Dfd* patterns the protopodite: the proximal part of the appendage including the endite [36]. *Tc-mxp* patterns the telopodite (the palp) and mutants of *Tc-mxp* possess legs instead of palps in both the maxillary and labial segments. These transformed appendages are attached to a protopodite that is unaffected by the loss of *Tc-mxp*[50,51].

**Figure 6:**
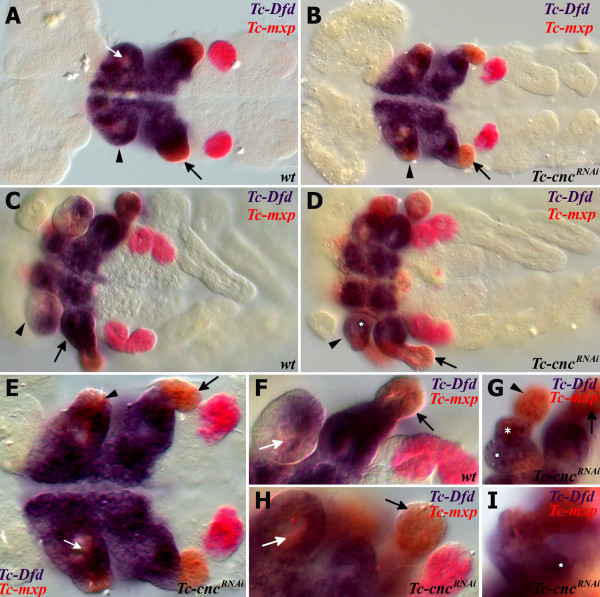
**Expression of the Hox genes *Tc-Dfd *and *Tc-mxp *in wild-type and *Tc-cnc***^***RNAi***^**embryos.** Knockdown of *Tc-cnc* by RNAi results in transformation of the mandibular appendage to maxillary identity and the expression of Hox genes in a similar manner to that seen in the maxilla. All views are ventral with anterior to the left. Expression of *Tc-Dfd* (blue) and *Tc-mxp* (red) was determined by *in situ* hybridization. Mandibular segment is indicated with an arrowhead, maxillary segment with a black arrow. Mesodermal expression of *Tc-mxp* is indicated with a white arrow. (**A,C,F**) Wild-type *Tribolium* embryos. (**B,D,E,G-I**) *Tc-cnc*^*RNAi*^ embryos. (**A**) Wild type germ band extending embryo. *Tc-mxp* is expressed in the developing maxillary and labial palps and the mesoderm in the mandibular segment (white arrow). (**B**) *Tc-cnc*^*RNAi*^ germ band extending embryo: *Tc-mxp* expression is present in the transformed mandibular appendage (arrowhead) in a telopodite domain consistent with the transformation of the mandible to maxillary identity. (**C**) Wild-type germ band retracting stage embryo. *Tc-Dfd* expression has retracted from the majority of the mandibular appendage. (**D**) *Tc-cnc*^*RNAi*^ embryo at a similar stage to C. *Tc-Dfd* expression is present in the transformed mandibular protopodite (star). *Tc-mxp* is expressed in the transformed mandibular appendage palp. (**E**) Higher magnification of the earlier germ band extending stage *Tc-cnc*^*RNAi*^ embryo shown in B. (**F**) Higher magnification of the gnathal appendages of a germ band retracting stage at a similar stage to **C**. (**G**, **H**, **I**) Higher magnification of the gnathal appendages of germ band retracting stage *Tc-cnc*^*RNAi*^ embryos. (**G**) *Tc-Dfd* is expressed throughout the transformed mandibular appendage, in the lacinia endite (star) and galea endite (asterisk). *Tc-mxp* is expressed in the palp (arrowhead) as well as the galea endite in a manner that is identical to the maxilla (arrow). (**H**) The mesodermal expression domain of *Tc-mxp* (white arrow) is observed in the transformed mandibular appendage. (**I**) *Tc-Dfd* is expressed throughout the maxilla, the rounded kink at the base of the maxilla is indicated (star).

As *Tc-cnc* RNAi results in a homeotic transformation of the mandible into a maxilla, we predicted that both the Hox genes responsible for patterning the maxillary appendage will be expressed in the maxillary pattern in the homeotically transformed appendage. It was found that this is indeed the case (see Figure 6).

In wild-type embryos, *Tc-Dfd* expression retracts from the mandibular limb bud (see Figure 6C,F). In *Tc-cnc*^*RNAi*^ embryos, the mandible is transformed to maxillary identity and *Tc-Dfd* expression is retained in the protopodite of this transformed appendage (see Figure 6D,G-I).

*Tc-mxp* is expressed in the maxillary and labial palps in wild-type embryos (see Figure 6A,C,F). In the maxillae, *Tc-mxp* is expressed in the distal part of the protopodite, including the galea endite. In the transformed mandibular appendage of *Tc-cnc*^*RNAi*^ embryos, *Tc-mxp* is expressed in the ectoderm of the ectopic palp as it is in the maxillary palp and also includes expression in the galea endite and distal protopodite (see Figure 6B,D,E,G,I).

*Tc-mxp* is expressed in the mesoderm of the mandibular appendages of wild-type embryos (white arrow in Figure 6A,F) [51]. Interestingly, this mesodermal expression of *Tc-mxp* is seen in *Tc-cnc* knockdown embryos (white arrow in Figure 6E,H). This suggests that there is *cnc* independent regulation of *Tc-mxp* in the mandibular limb bud. *Tc-cnc* is expressed in the ectoderm of the mandibular limb bud, and expression is weaker (or absent) in the mesoderm.

### *Tc-Dfd* activates the posterior ‘collar’ domain of *Tc-cnc* in the mandibular segment

Experiments performed on *Drosophila* have shown that *Dfd* does not activate *cnc* expression [52]. In order to investigate whether *Tc-Dfd* has any role in regulating *Tc-cnc* expression in *Tribolium,* we knocked down *Tc-Dfd* by parental RNAi and detected *Tc-cnc* expression via *in situ* hybridization.

**Figure 7:**
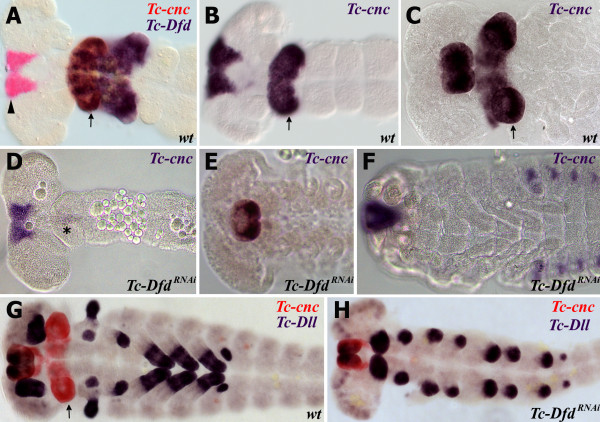
***Tc-Dfd *activates the posterior collar domain of *Tc-cnc *in the mandibular segment.** Gene expression was determined by *in situ* hybridization. (2**A-C**) *Tc-cnc* expression in wild-type embryos. Throughout embryogenesis, *Tc-cnc* expression consists of an anterior cap domain in the labrum (arrowhead) and a collar domain (arrow) in the mandibular segment. (**A**) *Tc-cnc* (red) and *Tc-Dfd* (blue) expression in a germ band extending embryo. (**B**) *Tc-cnc* expression (blue) in a germ band extending embryo at a similar but slightly earlier stage to (**A**). (**C**) *Tc-cnc* expression (blue) in later stage embryo prior to dorsal closure. (**D-F**) *Tc-cnc* expression in *Tc-Dfd*^*RNAi*^ embryos. In all stages, from germ band extending (**D**), germ band retracted (E) and during dorsal closure (F), the posterior domain of *Tc-cnc* is missing in the mandibular segment, whilst the anterior domain of *Tc-cnc* is expressed as normal showing that *Tc-cnc* is activated by *Tc-Dfd* in the mandibular segment. There is a faint stripe of *Tc-cnc* in the mandibular segment of (D) (asterisk), this may be due to partial knockdown effects.( **G**) Expression of *Tc-cnc* (red) and *Tc-Dll* (blue) in wild-type germ band retracting embryo.( **H**) Expression of *Tc-cnc* (red) and *Tc-Dll* (blue) in a *Tc-Dfd*^*RNAi*^ germ band extending embryo. The posterior domain of *Tc-cnc* is missing.

Surprisingly, we found that in *Tc-Dfd*^*RNAi*^ embryos the posterior collar domain of *Tc-cnc* expression is completely missing from all stages of embryo investigated, from germ band extending embryos through to stages where embryos are undergoing dorsal closure (Figure 7). The anterior cap domain of expression is unaffected. This shows that, unlike in *Drosophila, Tc-Dfd* is necessary for the activation of the posterior domain of *Tc-cnc* in the mandibular segment of *Tribolium*.

### *Tc-Dfd* activates *Tc-prd* expression in the mandible and maxillary segments

Brown *et al.* have shown that *Tc-Dfd* is required to pattern the mandible and the proximal part of the maxillary appendages In *Tc-Dfd* mutants, the mandible is transformed to antennal identity and the maxillae lose the endites whilst retaining the palp [36].

**Figure 8:**
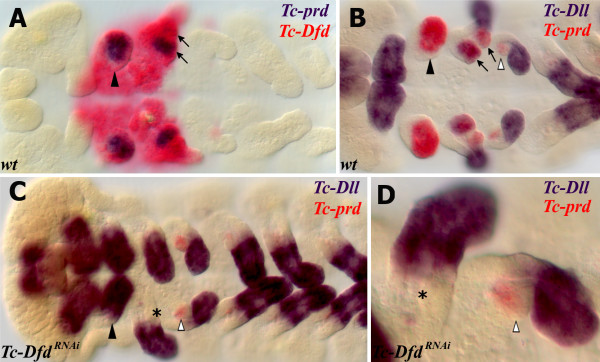
***Tc-Dfd *knockdown results in the loss of *Tc-prd *expression in the embryonic mandibular and maxillary segments.***Tc-Dfd* patterns the endites of the mandibular and maxillary segments. The mandibular segment appendage is marked with an arrowhead. The maxillary endites are marked with arrows in wild-type embryos (**A,B**). The labial endites are marked with white arrowheads. (**A**) Expression of *Tc-prd* (blue) and *Tc-Dfd* (red) in a wild-type germ band extending embryo. *Tc-prd* is expressed in the developing endites of the mandible and maxilla. *Tc-Dfd* is expressed in the mandibular and maxillary segments. (**B**) Expression of *Tc-prd* (red) and *Tc-Dll* (blue) in a wild-type germ band extending embryo. *Tc-prd* is expressed in the mandible, maxillary and labial appendages. *Tc-Dll* is expressed in the lacinea endite lobe. There is no *Tc-Dll* expression in the mandible (arrowhead). (**C,D**) Expression of *Tc-prd* (red) and *Tc-Dll* (blue) in a *Tc-Dfd*^*RNAi*^ germ band extending embryo. (**C**) The mandible has been transformed into an ectopic antenna, which expresses *Tc-Dll* (arrowhead) and lacks *Tc-prd* expression. There is no endite and no *Tc-prd* expression (asterisk) in the maxilla. The labial appendage has an endite (white arrowhead) marked with *Tc-prd* expression. (**D**) Enlargement of the maxilla and labial appendage shown in (**C**).

In order to further investigate the role of *Tc-Dfd* in patterning the gnathal appendages, we studied *Tc-prd* expression in *Tc-Dfd*^*RNAi*^ knockdown embryos. In *Tc-Dfd*^*RNAi*^ knockdown embryos, *Tc-prd* expression is lacking in both the transformed mandible (ectopic antennae) and the affected maxillary appendages (see Figure 8C,D). *Tc-prd* is still expressed in the developing labial endite. This result shows that *Tc-Dfd* is necessary for the activation of *Tc-prd* expression in the mandibular and maxillary segments and is further evidence that *Tc-Dfd* is required for development of the endites on these segments.

## Discussion

### The role of *Tc-cnc* in patterning the mandible of *Tribolium*

We sought to understand mandible patterning in a model arthropod that has a mandible with primitive characteristics. Our results show that *Tc-cnc* is required for specification of the identity of the mandibular segment of *Tribolium* and differentiates the mandible from a maxilla.

Knockdown of *Tc-cnc* transcripts by parental RNAi results in a homeotic transformation of the mandible into maxillary identity in *Tribolium* embryos and first instar larvae. The homeotic transformation is also evident in the changed expression of the genes *Tc-Dll* and *Tc-prd* (markers for the developing telopodite and endite of the maxilla) in knockdown embryos.

The Hox genes *Tc-mxp* and *Tc-Dfd* are required to pattern the maxillary appendage and do so in an additive manner, *Tc-Dfd* patterns the base of the appendage and *Tc-mxp* patterns the palp [36,51]. We show that in *Tc-cnc* knockdown embryos, *Tc-Dfd* and *Tc-mxp* are expressed in a maxilla like pattern in the transformed mandibular appendage.

We show that the ‘collar’ domain of *Tc-cnc* in the mandibular segment is activated by *Tc-Dfd* in *Tribolium.* The mandibular segment collar domain of *cnc* is not activated or regulated by *Dfd* or by any other Hox gene in *Drosophila*[52]. We also show that *Tc-Dfd* is necessary for the expression of *Tc-prd* in both the mandible and the maxilla.

**Figure 9:**
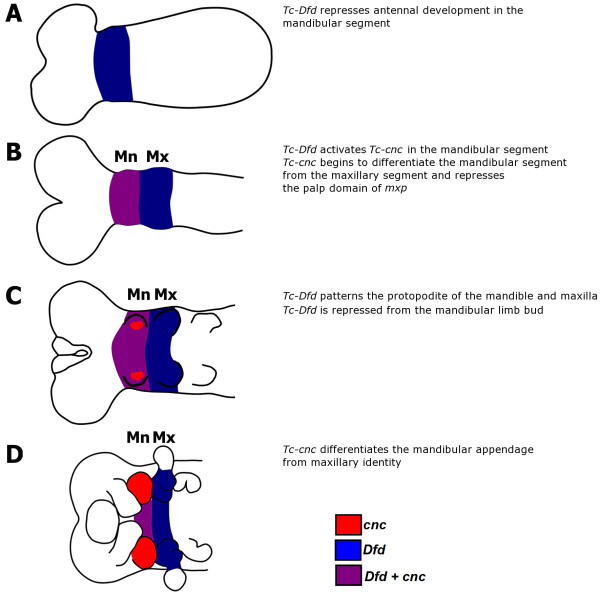
**A model of *Tc-cnc *and *Tc-Dfd *mandibular and maxillary patterning functions in *Tribolium castaneum.****Tc-Dfd* patterns both the mandible and maxillary segments. *Tc-Dfd* patterns the protopodites of these appendages. Note that the anterior ‘cap’ domain of *Tc-cnc* has been omitted from this scheme for clarity. *Tc-Dfd* expression is shown in blue, *Tc-cnc* expression is shown in red. *Tc-cnc* and *Tc-Dfd* expression is shown in purple. (**A**) *Tc-Dfd* is expressed in the mandibular and maxillary segments and patterns these segments. In the mandibular segment, *Tc-Dfd* represses antennal development. *Tc-Dfd* patterns the maxillary segment in conjunction with *Tc-mxp.* (**B**) *Tc-Dfd* activates *Tc-cnc* expression and together *Tc-cnc* and *Tc-Dfd* cooperate to pattern the mandibular segment. (**C**) *Tc-Dfd* patterns the protopodite of the mandibular and maxillary appendages in germ band extending embryos, but is repressed from the mandibular limb bud as it develops. (**D**) In germ band retracting stage embryos, *Tc-cnc* has differentiated the mandibular appendage from maxillary identity.

Based upon the results of this and previous studies we present a model for the roles of these genes in mandible patterning in *Tribolium* (see Figure 9).

### The role of *Tc-cnc* in patterning the labrum of *Tribolium*

The deletion of the labrum in *Tc-cnc*^*RNAi*^ embryos is consistent with the loss of the cap domain of *Tc-cnc* expression in the labrum. The labrum is a structure of considerable interest as it is shared by all extant groups of euarthropods whilst its evolution and development remain controversial. The labrum has appendage-like characteristics and may have evolved from a fused pair of appendages, for example from structures homologous to the anterior antennae of lobopods [53]. However, unlike all other paired arthropod appendages, the labrum is not associated with a segment and may have a different origin [54].

### Comparisons with *Drosophila*

There are many similarities between *Tribolium* and *Drosophila* in the expression patterns of genes in the mandibular and maxillary segments and in how these segments are patterned. In both insects *Dfd* and *cnc* are both required to pattern the mandibular segment. *cnc* is required for the patterning of labral derived structures and the differentiation of the mandible from maxillary identity. *cnc* represses *Dll* expression in the mandibular segment*.*

The Hox genes *Dfd* and *pb/Tc-mxp* are also expressed in similar proximal and distal domains respectively in the maxillary segment limb bud or gnathal lobe as are *prd* and *Dll. Dfd* patterns proximal structures that are derived from the maxillary lobe or limb buds. In both species, *Dfd* activates the proximal domain of *Dll*[24,25,36]. *Tc-Dfd* activates the maxillary *prd* domain in both *Drosophila,* and also, as we have shown in this study, in *Tribolium*[55,56].

There are nevertheless differences in the patterning of the mandibular and maxillary segments between *Tribolium* and *Drosophila.* In *Drosophila,* loss of *cnc* function does not result in a full homeotic transformation of the mandibular gnathal lobe to maxillary identity, rather*,* the mandibular gnathal lobe is transformed into just the proximal part of the maxillary gnathal lobe [24]. This is in contrast to *Tribolium* where loss of *Tc-cnc* function results in a complete transformation of mandible to maxillary identity.

In addition to the activation of *Tc-cnc* in the mandibular segment by *Tc-Dfd*, another difference between *Drosophila* and *Tribolium* is the regulation of *collier (col)* by *cnc.* The anterior mandibular expression of *cnc* is upstream of *col* in *Drosophila* and both genes are required to pattern the hypopharyngeal lobes [57-59]. In *Tribolium,* which does not have hypopharyngeal lobes, it has been recently shown that *Tc-cnc* is not activated by *Tc-col*[60]*.*

### The role of *cnc* as a repressor of maxilla patterning Hox genes

While we have shown that *Tc-cnc* patterns the mandible and differentiates the mandible from a maxilla, the precise role that it has in patterning the mandibular segment is not clear. The many similarities in the patterning function of *cnc* in *Tribolium* and *Drosophila* suggest that the molecular functions of Cnc protein revealed by experiments in *Drosophila* may be similar in *Tribolium.*

Research in *Drosophila* has demonstrated the role of *cnc* as a repressor of Hox gene function in the mandibular segment [23,24]. *cnc* has been shown to repress *Dfd* transcription and Dfd protein activity in the anterior mandibular segment in *Drosophila*[23,24]*.* There is co-expression of *cnc* and *Dfd* in the posterior of the mandibular segment, indicating that some mandibular expression of *Dfd* is not affected by the presence of *cnc*[23,24]. *Dfd* has also been shown to repress *pb* in the ectoderm of the mandibular segment in *Drosophila*[61].

As the dynamics of *Tc-Dfd* expression in *Tribolium* resemble the dynamics of *Dfd* expression in *Drosophila,* with initial coexpression followed by subsequent repression of *Tc-Dfd* in a part of the mandibular segment, it seems likely that a similar situation is occurring in *Tribolium.*

We have shown that *Tc-cnc* is necessary for both the repression of *Tc-Dfd* expression in the mandibular limb bud and the repression of the ectodermal palp domain of *Tc-mxp* in the developing mandibular limb bud. However, further research is needed to determine whether *Tc-cnc* has a direct functional role in the repression of these Hox genes.

### The possible role of *Tc-cnc* as a direct activator of mandible patterning genes

In *Drosophila,* several lines of evidence suggest that Cnc functions as an activator, activating mandibular segment specific patterning genes and thereby indirectly repressing Hox genes [23]. *cnc* also patterns some mandibular segment derived structures independently of *Dfd*. Ectopic activation of *cnc* in *Drosophila* embryos results in ectopic hypopharyngeal lobe derived structures [23]. Although the hypopharyngeal lobes have been thought to derive from the intercalary segment, it has recently been shown that they are in fact derived from the mandibular segment [37]. This result indicates that *cnc* is in fact necessary and sufficient to pattern some mandibular segment derived structures suggesting that *Tc-cnc* may directly activate mandible patterning genes in *Tribolium*.

### Conserved expression of *cnc*, *Dfd* and *pb* in mandibulate arthropods

Comparison of the expression of *cnc* homologs in mandibulates suggests that both functions of the labral patterning anterior ‘cap’ domain and the mandible patterning posterior ‘collar’ domain are conserved in mandibulate arthropods. Species that have been studied in addition to *Drosophila* and *Tribolium* include the cricket *Acheta domestica,* the milkweed bug *Oncopeltus fasciatus,* and the firebrat *Thermobia domestica*[39,40]. Outside insects, only one species has been studied to date, the myriapod *Glomeris marginata*, which also shows expression in a cap and a mandibular collar [38].

The expression patterns of orthologs of *Dfd* and *pb* are also conserved in other mandibulates suggesting that patterning of the maxilla may also be conserved. *Dfd* is expressed in the mandible and maxilla bearing segments in the majority of mandibulates and expression is stronger in the protopodite than in the palps of maxillary appendages [36,39,62-66]. There is loss of *Dfd* expression in the mandibular limb bud across mandibulates, as in *Tribolium* and *Drosophila*[24,35,65,66]. Expression of *pb* is conserved in the telopodites of these maxillary appendages [39,65,67].

In an onychophoran, the closest extant outgroup to the Arthropoda, a homolog of *Dfd* is expressed in the proximal region of each walking limb bud [68] suggesting that *Dfd* expression in the base of the mandibular and maxillary limbs may be the primitive condition in the Arthropoda.

### *cnc* and the evolution of the mandible from a maxilla-like precursor

The manner in which *cnc* differentiates the mandible from maxillary identity may ultimately provide clues about how the mandible has evolved from a maxilla-like precursor in the stem lineage of mandibulate arthropods.

**Figure 10:**
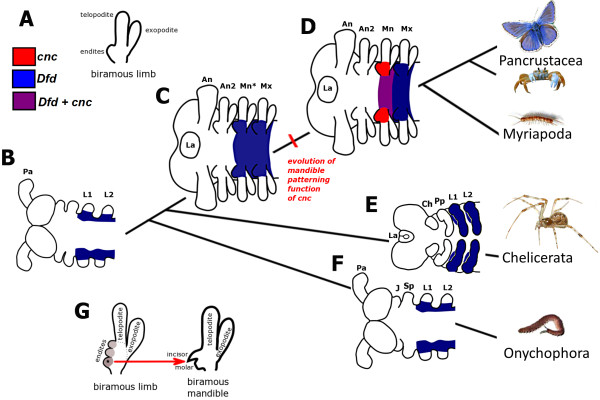
**Hypothetical evolution of the mandible patterning function of *cnc *in embryos in the stem lineage of the mandibulate arthropods.** (**A**) The post-antennal limbs of stem-lineage mandibulates are serially homologous biramous limbs with multiple endites, represented here as a single lobe for clarity, on the medial part of the protopodite. (**B**) Hypothetical expression of *Dfd* in a lobopod, the ancestor to all arthropods (and closely related taxa such as the Onychophora) based on expression of *Dfd* in an onychophoran [68]. Here, *Dfd* is expressed proximally in monopodial limbs with the anterior limit at the segment homologous to the first leg segment (L1) of chelicerates and onychophorans. (**C**) Hypothetical expression of *Dfd* in a hypothetical non-mandibulate ancestor to Mandibulata. (**D**) Expression of *cnc* and *Dfd* in a hypothetical ancestor to the mandibulate arthropods (Pancrustacea and Myriapoda). The mandibular segment identity is specified by *cnc.* We hypothesize that the mandible patterning function of *cnc* evolved in the stem lineage of the mandibulate arthropods. The mandibular and maxillary segments of mandibulates are homologous to the first and second leg segments of chelicerates and onychophorans. (**E**) Expression of *Dfd* in chelicerates based upon *Dfd* expression in spiders. There are two homologs of *Dfd* in spiders, both of which are expressed in the L1 to L4 segments. (**F**) Expression of *Dfd* in an onychophoran. (**G**) The mandibular gnathal edge, consisting of an incisor and molar, most likely evolved from the proximal endite (star) on the primitive biramous limb present in species such as *Martinssonia*. The other more distal endites were lost at some point. Labels are: antenna (An), first leg (L1), jaws (J), labrum (La), mandible (Mn), maxilla (Mx), maxilla-like mandible precursor (Mn*), primary antenna (Pa), second antenna (An2), second leg (L2), slime papilla (Sp).

A study of the fossil record shows that the mandible has evolved from a particular type of jointed appendage, the biramous limb (see Figure 10A). In the ancestor to the arthropods, the primitive post-antennal limbs were similar in structure [12]. As stem lineage arthropods diverged during the Cambrian, post-antennal biramous limbs diverged from the primitive biramous limb structure. The likely precursor to the mandible was a maxilla-like appendage, with numerous well-defined endites similar to those present on other post-antennal segments (see Figure 10G). Such a maxilla-like second post-antennal limb is present in numerous ‘crustaceamorph’ stem lineage mandibulate arthropods like *Martinssonia elongata* and the Phospatocopida [2,11,69,70].

We hypothesize that, in the stem lineage to the mandibulate arthropods, *Dfd* patterned the base of the ancestral monopodial limb (see Figure 10B) and the protopodite of the primitive biramous gnathal appendages (see Figure 10C). At some point in the stem-lineage leading to the mandibulate arthropods, *cnc* acquired a new role patterning the mandibular segment: differentiating the mandibular endite and protopodite from those of the maxilla resulting in the mandibular gnathal edge (see Figure 10D).

The mandible has probably evolved from a biramous maxilla-like precursor by modification of the most proximal endite to form the characteristic mandibular gnathal edge whilst, at least primitively, retaining both the telopodite palp and the exopodite (see Figure 10G).

### The role of *cnc* homologs in chelicerates and onychophorans

To test the idea that the function of *cnc* evolved to pattern the mandible in the lineage leading to the mandibulates, it is necessary to study *cnc* homologs in outgroups to the Mandibulata with the prediction that it does not have a comparable role in patterning the segment homologous to the mandibular segment (the first leg segment in chelicerates).

The homologous segment to the mandibular segment in the chelicerates and the onychophorans is the first leg segment and homologs of *Dfd* are expressed in this segment (see Figure 10E,F) [4,5,68]. In these groups there is no obvious differentiation between the first leg appendage and the second leg appendage (maxilla homolog). It is therefore not obvious what role a ‘collar’ domain of *cnc* would perform in chelicerates or onychophorans.

Although the expression of *cnc* is not known in non-mandibulate arthropods, expression of chelicerate anterior Hox genes such as *Dfd* and *pb* are different in several respects to the conserved expression of these genes in mandibulate arthropods [62]. This suggests that the conserved expression of Hox genes in the mouthparts of the mandibulate arthropods is a synapomorphy for the Mandibulata [5,71,72].

The closest related outgroup of the Mandibulata in which a *cnc* homolog has been investigated is the nematode *Caenorhabditis elegans*. The *C. elegans cnc* homolog, Skn1, has been shown to have developmental role in patterning mesoderm and endoderm derived structures [73,74]. One important, non-developmental role of *cnc* (and its homologs across Bilateria) that *has* been studied in some detail is its role in xenobiotic and oxidative stress responses [49,75-77]. This role has been discovered in diverse organisms and is likely to be present both in mandibulates and in closely related outgroups to the Mandibulata such as the chelicerates.

## Conclusions

Our study is the first functional investigation of some of the genes necessary specifically to pattern the mandible of an arthropod species with a canonical mandible in which the gnathal edge is made up of the incisor and molar processes.

Using parental RNAi to knockdown gene transcripts in *Tribolium*, we show that *Tc-cnc* is required for specification of the identity of the mandibular appendage and differentiates it from maxillary identity. Analysis of gene expression by *in situ* hybridization shows that *Tc-cnc* is required for the repression of the maxillary expression domains of the Hox genes *Tc-mxp* and *Tc-Dfd,* which pattern the maxilla. We also show that *Tc-cnc* is necessary for the formation of the labrum. The mandible differentiating function of *Tc-cnc* is similar to the role of *cnc* in *Drosophila* in patterning the mandibular segment; in both beetle and fly, *cnc* and *Dfd* cooperate to specify mandibular identity. One significant difference is that *Tc-cnc* is activated by *Tc-Dfd* in the mandibular segment in *Tribolium* whereas *cnc* is activated independently of *Dfd* in *Drosophila.*

Similar expression patterns of *cnc, Dfd* and *pb* homologs in other mandibulate arthropods suggests that the functions of these genes are conserved, that *cnc* also differentiates the mandible from the maxilla in these species and that *cnc* evolved a mandible patterning function in the lineage leading to the mandibulates and possibly acts in conjunction with *Dfd* to achieve this.

To show that *cnc* has a conserved role in patterning the mandible across Mandibulata requires study of the function of *cnc*, or at the very least additional expression data, in more representatives of the mandibulate arthropods. In particular, expression data are lacking from any crustacean species.

## Competing interests

The authors declare that they have no competing interests.

## Authors’ contributions

JFC and MJT conceived and designed the study. JFC collected the data and JFC and MJT analyzed the results. JFC and MJT drafted the manuscript and approved the final manuscript for submission.
